# Fatalities in Oil and Gas Extraction Database, an Industry-Specific Worker Fatality Surveillance System — United States, 2014–2019

**DOI:** 10.15585/mmwr.ss7208a1

**Published:** 2023-09-01

**Authors:** Kaitlin C. Wingate, Alejandra Ramirez-Cardenas, Ryan Hill, Sophia Ridl, Kyla Hagan-Haynes

**Affiliations:** 1 Western States Division, National Institute for Occupational Safety and Health, CDC

## Abstract

**Problem/Condition:**

The U.S. oil and gas extraction (OGE) industry faces unique safety and health hazards and historically elevated fatality rates. The lack of existing surveillance data and occupational safety and health research called for increased efforts to better understand factors contributing to worker fatalities in the OGE industry. This report describes the creation of the Fatalities in Oil and Gas Extraction (FOG) database, presents initial findings from the first 6 years of data collection (2014–2019), highlights ways that FOG data have been used, and describes the benefits and challenges of maintaining the surveillance system.

**Period Covered:**

2014–2019.

**Description of System:**

In 2013, the National Institute for Occupational Safety and Health (NIOSH) created the FOG database, a surveillance system comprising an industry-specific worker fatality database. NIOSH researchers worked with OGE partners to establish inclusion criteria for the database and develop unique database variables to elucidate industry-specific factors related to each fatality (e.g., phase of operation, worker activity, and working alone). FOG cases are identified through various sources, such as Occupational Safety and Health Administration (OSHA) reports, media reports, and notifications from professional contacts. NIOSH researchers compile source documents; OGE-specific database variables are coded by multiple researchers to ensure accuracy. Data collection ceased in 2019 because grant funding ended.

**Results:**

During 2014–2019, a total of 470 OGE worker fatalities were identified in the FOG database. A majority of these fatalities (69.4%) were identified from OSHA reports and Google Alerts (44.7% and 24.7%, respectively). Unique database variables created to characterize fatalities in the OGE industry (i.e., phase of operation, worker activity, working alone, and working unobserved) were identified in approximately 85% of OGE worker fatality cases. The most frequent fatal events were vehicle incidents (26.8%), contact injuries (21.7%), and explosions (14.5%). The event type was unknown among 5.7% of worker fatalities. Approximately three fourths of fatalities identified through the FOG database were among contractors. Approximately 20% of cases included workers who were working alone.

**Interpretation:**

The FOG database is a resource for identifying safety and health trends and emerging issues among OGE workers (e.g., exposure to hydrocarbon gases and vapors and fatalities resulting from cardiac events) that might not be available in other surveillance systems. The FOG database also helps researchers better identify groups of workers that are at increased risk for injury in an already high-risk industry. Challenges exist when maintaining an industry-specific surveillance system, including labor-intensive data collection, the need for researchers with substantial knowledge of the industry, delays in access to timely data, and missing source file data.

**Public Health Actions:**

Continued surveillance of worker fatalities in the OGE industry is recommended to help identify new safety and health hazards and guide research and prevention activities. Industry, academic institutions, and government can use findings from the FOG database to identify factors contributing to fatal injuries in OGE and develop interventions to improve worker safety and health. The findings in this report also can be used by other industries with high fatality rates to support the development of worker fatality surveillance systems.

## Introduction

Oil and gas extraction (OGE) work environments and corresponding safety and health hazards are unique, complex, and lacking in research by public health and occupational health communities (*1*–*3*). OGE workers face elevated fatal occupational injury rates that are historically seven times higher than for all U.S. workers (*4*). OGE workers have physically demanding jobs, are exposed to hazardous chemicals and flammable and toxic gases, experience long commutes, engage in shift work, and work outdoors in all environmental conditions (*1*,*2*,*5*,*6*).

OGE workers are responsible for a wide range of operations, including constructing oil and gas well sites, drilling and fracturing the wellbore, and bringing fluids to the surface for transport to refineries. Workers also provide maintenance and monitoring of well sites to ensure ongoing oil and gas production meets the energy demands of U.S. citizens and for use in the development of multiple other types of consumer products. The U.S. OGE industry falls within the mining, quarrying, and oil and gas extraction sector (coded 21 by the North American Industrial Classification System [NAICS]) and generally is grouped into three subsectors: operators who develop and operate leased properties (211), drilling contractors who drill oil and gas wells and perform well control activities (213111), and well servicing contractors who provide specialized support services for existing wells (213112) (*7*). Although the OGE workforce comprised approximately 70% of the mining sector workforce in 2019, it accounted for approximately 82% of all fatalities (*8*,*9*). Transportation incidents account for the largest proportion of OGE worker fatalities (*8*). Efforts to understand and respond to oil and gas worker injuries and fatalities require close industry, state, and federal government collaboration, in part because of the lack of an OGE industry safety and health standard and the ununionized circumstances of the industry. However, OGE workers provide an indispensable service to the U.S. economy as demand for oil and gas continues domestically and globally (*10*,*11*).

Researchers from the National Institute for Occupational Safety and Health (NIOSH) Oil and Gas Extraction Sector Program conducted studies using data collected from the Bureau of Labor Statistics (BLS) Census of Fatal Occupational Injuries (CFOI) to better understand the factors placing OGE workers at higher risk for fatality (*12*). These researchers found a statistically significant decrease in the rate of occupational fatalities during 2003–2013, a period when the workforce size doubled; drilling contractors were identified as having the highest fatality rates (*4*). An analysis of motor vehicle-related fatalities among OGE workers illustrated that well servicing contractors had the highest rates of crash death and highlighted speeding and lack of seat belt use as contributing factors for the fatal crashes (*13*). An analysis of offshore fatalities from CFOI also found that transportation events (e.g., aircraft and water vehicle events) accounted for approximately one half of all fatalities and identified inclement weather and mechanical failures as common contributing factors (*14*). Recommendations from these studies called for enhanced surveillance efforts to better understand the contributing factors to OGE worker fatalities and guide intervention strategies to be implemented by industry safety and health professionals (*3*,*4*).

CFOI collects worker demographic attributes and circumstances of worker fatalities as a census of worker fatalities in the United States and is considered the leading source of information on occupational fatalities (*12*). However, CFOI was not designed to include factors that are industry specific and might provide critical insight into a certain industry. OGE workplace environments vary substantially, including diverse operations, types of equipment used, activities tasked to workers, and health and safety hazards. In addition, CFOI does not report on fatalities that occur during a worker’s commute unless the commute was outside of the worker’s geographic or temporal routine, the location varied because of the itinerant nature of the work, or the transportation was provided by the employer (*15*). This determination is consistent with policies of the Occupational Safety and Health Administration (OSHA) and workers compensation agencies, where injuries that occur while a worker is commuting are not considered work related (*16*). Commuting is of specific concern for the OGE industry because much of the work activity occurs in remote locations, requiring long commutes to a work site. Lastly, CFOI considers heart attacks as illnesses and excludes these deaths from the database (*12*). Exposure to toxic gases and vapors is a concern for OGE safety and health experts because acute exposures could mimic or induce cardiac events. No available data source exists that systematically collects and examines occupational cardiac fatalities in the United States. One high-risk industry with a worker fatality surveillance system (i.e., structural firefighters) includes deaths resulting from heart attacks if the worker became ill within 24 hours of engaging in nonroutine, stressful, or strenuous physical activity while on duty (*17*).

The National Occupational Research Agenda (NORA) OGE Sector Council was created in 2008 to stimulate innovative research and improve safety and health practices and includes participation from industry, trade organizations, academic partners, and government (*18*). NIOSH researchers shared oil- and gas-specific CFOI findings with the NORA OGE Sector Council, which responded with questions about the circumstances of fatalities specific to OGE operations, such as the phase of well development and activity being performed. NIOSH researchers recognized the complexity of OGE worksites, including temporary worksites, multiple phases of well development, remote locations, multiemployer worksites with multiple layers of contractors and subcontractors, specialized heavy moving equipment, high employee turnover rates, highly flammable and potentially toxic gases on-site, and the cyclical nature of the industry. Location also was an important consideration, because OGE activity and exposures vary across different basins (regions rich in sediment rock allowing for oil and gas production) (*19*). From an occupational safety and health perspective, CFOI data provided little insight into the stage of well development, the specific activities and equipment, and other factors that characterized OGE worker fatalities. Although researchers can request access to restricted CFOI data, CFOI data that are publicly available are aggregated to protect confidentiality, further adding to the limitations of the data source.

An additional challenge to existing OGE fatality data was the omission of fatalities among contractors working in support of the OGE industry but who are employed by a company that is not included in one of the three OGE NAICS codes. A report of offshore OGE fatalities indicated that approximately one third of fatalities were among workers employed in industries other than those corresponding to three standard NAICS codes (*14*). NORA OGE Sector Council members reported that certain transportation contractors (e.g., water or crude oil haulers) worked in OGE only during energy booms when OGE activity was high but worked in other industries when oil prices were low. The concern for these workers was their lack of knowledge about OGE industry hazards because of their transient nature in oil fields. Discussions with the council prompted the idea to develop a pilot OGE worker fatality surveillance system to provide additional insights into OGE worker fatalities that were not available through CFOI. The council wanted to have actionable insights on key OGE worksite factors that could be used in safety and health interventions. The worker fatality surveillance system was created in 2013 and comprised the Fatalities in Oil and Gas Extraction (FOG) database, which the most current NORA OGE Sector Research Agenda highlights as an important source of information (*18*).

This report provides an overview of the steps in creating the FOG database, initial findings from 6 years of data collection (2014–2019), the ways that FOG data have been used over the past 8 years, and the benefits and challenges of maintaining an industry-specific worker fatality surveillance system. These findings will help characterize OGE workers and identify factors contributing to fatal injuries across the industry, enabling industry, academia, and government to develop additional initiatives to improve OGE worker safety and health. Other industries with high fatal occupational injury rates (e.g., mining and construction) (*8*) also can use this report to support the development of an industry-specific worker fatality surveillance system.

## Methods

During 2013–2014, researchers from NIOSH’s Oil and Gas Extraction Sector Program (i.e., epidemiologists, an industrial hygienist, and a toxicologist) met with members from the NORA OGE Sector Council to examine the data missing from available data sets on OGE worker fatalities and to develop the inclusion criteria and database variables for the FOG database. These meetings occurred in person during the biannual NORA OGE Sector Council meetings and virtually as needed. To identify additional industry-specific variables, researchers also reviewed a convenience sample of English-language articles that included worker safety or described activities common in the industry from OnePetro (*20*), an online library of technical literature for the oil and gas industry, and the International Association of Drilling Contractors (IADC) Incident Statistics Program, a database of safety and accident information among drilling contractors. This activity was reviewed by CDC and was conducted consistent with applicable federal law and CDC policy.*

### Variable Creation

Industry and government representatives’ discussion at NORA OGE Sector Council meetings formed the basis for determining which variables to incorporate into the FOG database. Details about the incident (e.g., date, location, and circumstances surrounding the incident) and details specific to each fatally injured worker (e.g., worker demographics, type and size of worker’s employer, and cause of the fatality) were deemed necessary. Other variables included in the FOG database were consistent with other fatality surveillance systems (e.g., worker demographics).

The event type variable identified how the fatal incident occurred. These codes were aligned closely with the BLS Occupational Injury and Illness Classification System (OIICS) event type codes (*21*); however, slight additions and modifications were required to fit the needs of describing fatalities in OGE. First, the FOG database includes all types of fatalities on the worksite, including cardiac events. Although OIICS is designed to identify injury event types, codes for cardiac events were not included. Second, specific types of exposures were important to highlight in OGE but were not identified as an event type in OIICS.

Variables unique to the OGE industry were designed in consultation with NORA OGE Sector Council members to determine and refine response categories. One OGE-specific variable was the stage of well development during which the incident occurred. The variable was titled “Phase of Operation” and included the distinct stages or processes that occur throughout oil and gas well development and ongoing maintenance. A second OGE-specific variable was OGE worker activity, which included activities common to OGE and was designed to allow for up to four activities for an incident because of the complex nature of OGE work sites. Other variables included whether the worker was alone or unobserved at the time of the incident and a worker’s years of work experience in the oil field. Approximately 100 FOG database variables were included in the database (Supplementary Table 1, https://stacks.cdc.gov/view/cdc/131261).

In 2018, the event type, phase of operation, and activity variables were reviewed and updated by NIOSH oil and gas researchers to better reflect the OGE circumstances of fatalities during 2014–2017. An updated set of variable codes (FOG 2.0) was used to code fatalities during 2018–2019 and also was used to update codes for fatalities during 2014–2017 (Supplementary Table 2, https://stacks.cdc.gov/view/cdc/131261). FOG data are not currently being collected beyond 2019 because grant funding ended.

### Case Ascertainment

**FIGURE 1:**
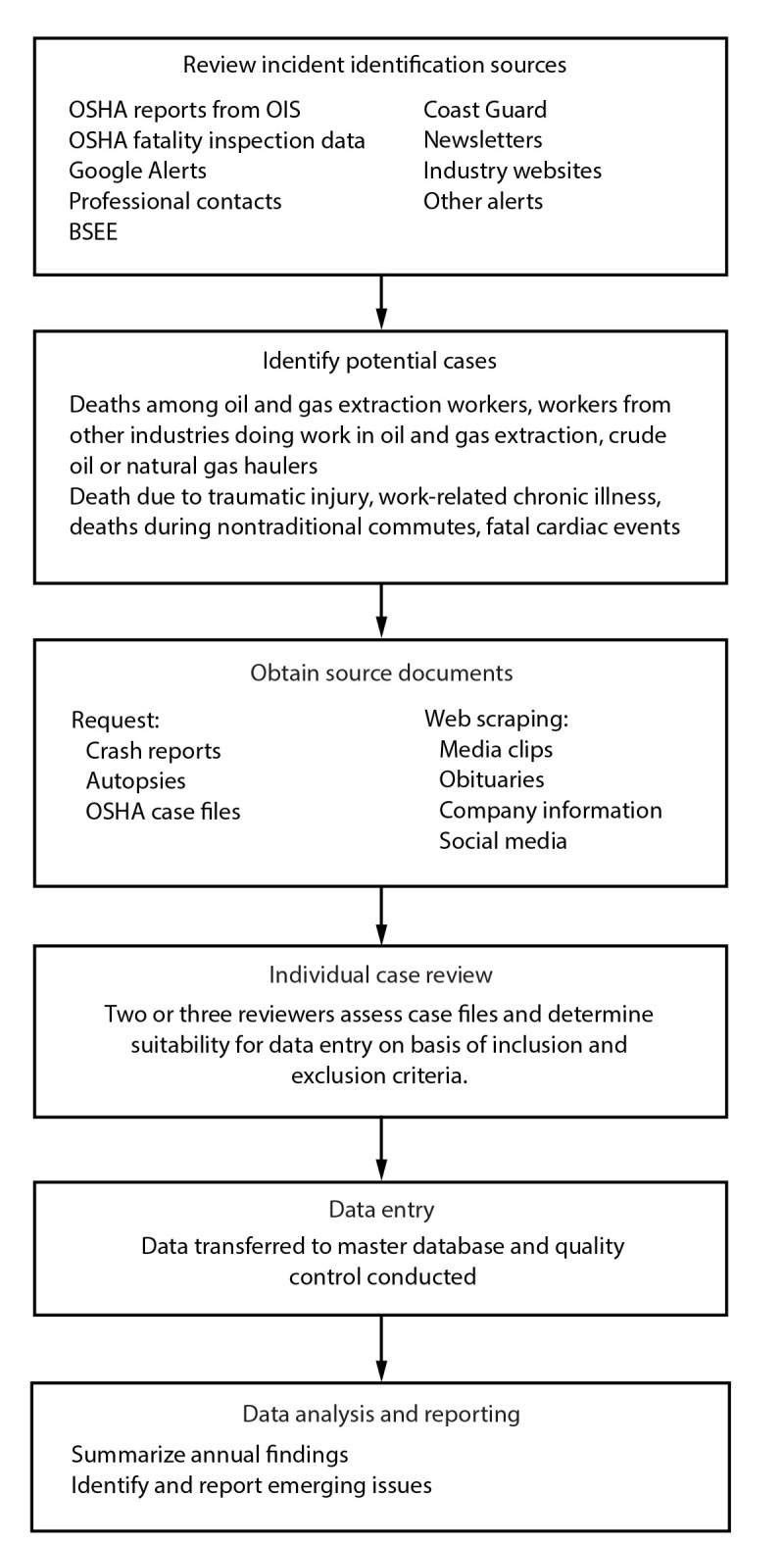
Case ascertainment, data collection, analysis, and reporting — Fatalities in Oil and Gas Extraction database, 2014–2019 **Abbreviations:** BSEE = Bureau of Safety and Environmental Enforcement; OIS = OSHA Information System; OSHA = Occupational Safety and Health Administration.

Information included in the FOG database is collected using a multistep process, which begins with identifying potential cases using various sources (Figure 1). Fatalities occurring because of a work-related incident must be reported to OSHA within 8 hours after the death, including deaths attributable to heart attacks (*22*). Multiple potential FOG cases are identified from the OSHA Information System through periodic requests for reports containing fatalities for NAICS codes 211, 213111, and 213112 and 237310 (i.e., highway, street, and bridge construction, which includes oil field road construction) or through the OSHA Fatality Inspection Database (*23*). Other sources used to identify cases include Google Alerts,^†^ notifications from professional contacts (i.e., OGE industry health and safety professionals), U.S. Bureau of Safety and Environmental Enforcement (BSEE) panel investigation reports (*24*), U.S. Coast Guard incident investigation reports (*25*), newsletters (e.g., https://jordanbarab.com/confinedspace), industry websites (e.g., https://www.roughneckcity.com), and other sources.

### Inclusion and Exclusion Criteria

The FOG database contains worker fatalities resulting from an incident that occurred during onshore or offshore OGE operations. The fatality could have occurred 1) on-site during exploration, site preparation, drilling, completing, servicing and ongoing operation of oil and gas wells in the United States; 2) during transport of OGE equipment, water, or processed fluids; 3) during OGE work-related travel; or 4) during nontraditional commutes (i.e., when traveling as a crew, with a paid driver, or >90 minutes or >50 miles each way).

The fatally injured worker must have been engaged in work for the OGE industry at the time of the incident. Although there are no specific inclusion criteria based on NAICS codes, a majority of fatalities from codes 211, 213111, and 213112 meet the inclusion criteria. Fatalities with these NAICS codes that did not meet the inclusion criteria had incorrect NAICS codes because of data entry errors. Workers employed by a company outside one of the three OGE NAICS codes were included if it could be confirmed that they were engaged in OGE activities.

The worker’s death must have been caused by a fatal injury or an onset of illness that occurred at work or was a direct result of work-related activities (e.g., traumatic injuries, work-related chronic illnesses, deaths during nontraditional commutes, and fatal cardiac events that began at work or were triggered by work activities). Excluded from the FOG database are workers with nonfatal injuries and illnesses and fatalities of workers in the midstream or downstream sectors of the oil and gas industry.

### Data Collection, Extraction, and Coding

After potential cases are identified, source documents are collected. If the case was investigated by OSHA, the case file is requested from the assigned OSHA area office after the investigation is complete, which can take up to 6 months. OSHA case files can include citations, violation worksheets, inspection summaries, inspection notes, police reports, emergency responder reports, medical examiner or coroner reports, toxicology results, and crash reports. If the case was not investigated by OSHA, the research team will work with local and state governments to locate each of the independent elements that an OSHA case file comprises, if available. Researchers also extract information from other sources, including news reports, media articles, obituaries, social media (e.g., LinkedIn, Facebook, and GoFundMe), and websites identifying company information (e.g., Manta, ZoomInfo, and the U.S. Department of Transportation Safety and Fitness Electronic Records System Company Snapshot).

All source documents for each fatal incident are compiled into a FOG case file. Two NIOSH researchers independently review each case file to determine whether the incident meets the inclusion criteria, note any items of interest, and code the key FOG database variables (i.e., activities, phase of operation, event type, industry group, working unobserved, working alone, age, years in the oil field, and other contributing or human factors). The researchers meet to discuss each case and reach consensus on the coded database variables. If consensus cannot be reached, a third researcher reviews the FOG case to help reach consensus. When consensus has been reached for each case and all database variables, a Fatality Information Form is completed for each fatality by a NIOSH researcher (Supplementary Appendix 1, https://stacks.cdc.gov/view/cdc/131261). Certain database variables require additional steps to complete (e.g., address details are used to identify the corresponding oil and gas basin and latitude and longitude coordinates are estimated if not directly provided). These data are entered into the database, and quality control is performed by checking for missing and inconsistent values to identify data entry errors and improve completeness. FOG database variables created with branching logic are reviewed to ensure that follow-up values are either blank or complete depending on the parent variable. Categorical database variables are compared with the corresponding numerical value, if available, to ensure accuracy.

### Texas Department of Transportation Crash Records Information System

Data sources such as OSHA reports often do not identify motor vehicle crashes, because fatalities resulting from motor vehicle crashes that occurred on a public street or highway are not required to be reported to OSHA (*22*). Because transportation incidents are the leading cause of occupational fatalities among OGE workers, NIOSH researchers developed a pilot program to test the ability of the Texas Department of Transportation Crash Records Information System (CRIS) to identify fatal crashes involving OGE workers. CRIS is an automated database of reportable motor vehicle traffic crashes received by the Texas Department of Transportation (*26*). To identify potential OGE worker fatalities during 2017–2019, CRIS was filtered to only display fatal events occurring in counties with OGE industry activity as outlined by the Railroad Commission of Texas (*27*) and involving persons aged ≥18 years. The remaining crash reports were reviewed by a researcher to determine whether the vehicle owner or lessee was an OGE company and if the fatally injured person was an OGE worker. Only fatal crashes occurring in vehicles owned by OGE companies were included in this pilot component of the FOG database.

## Analysis

Descriptive statistics were generated for this report using R (version 4.2.0; R Foundation). The percentage completeness of select FOG database variables was calculated. To determine the success of the FOG database in identifying occupational injury fatalities in OGE, the number of worker fatalities included in FOG, excluding those attributable to cardiac events, was compared with the number of fatalities identified in CFOI. Rates of worker fatalities included in FOG per 100 active rotary rigs were calculated using average annual active rotary rig counts from Baker Hughes (*28*). Descriptive statistics were generated for the following FOG database variables: data source, year, basin, age, sex, race and ethnicity, phase of operation, industry group, event type, activity, working alone, and offshore. Certain responses with multiple subtypes were grouped to preserve the anonymity of the fatally injured worker and for table and chart visualization purposes.

The mean age of worker fatalities was calculated. A map was created using ArcGIS Pro (version 2.9.3; Environmental Systems Research Institute) plotting the coordinates of each fatality and U.S. basins using data from the U.S. Energy Information Administration (*29*). Descriptive statistics were generated for worker fatalities identified from CRIS for the following FOG database variables: industry group, age, crash type, vehicle type, seat belt used, ejected from vehicle, and time of day.

## Results

### Data Source for Fatality Identification and Variable Completeness

During 2014–2019, a total of 470 OGE worker fatalities were identified in the FOG database (Table 1). The most common data sources in which worker fatalities were first identified were OSHA report data (44.7%), Google Alerts (24.7%), professional contacts (7.7%), and other media (7.0%).

**TABLE 1:** Number of fatalities and data source of initial case identification — Fatalities in Oil and Gas Extraction database, 2014–2019

Source	Fatalities No. (%)
OSHA report data	210 (44.7)
Google Alerts	116 (24.7)
Professional contacts	36 (7.7)
Other media	33 (7.0)
OSHA weekly reports	20 (4.3)
OSHA fatality inspection data	15 (3.2)
OSHA or NIOSH clips	5 (1.1)
BSEE	2 (0.4)
USCG	2 (0.4)
Newsletter	1 (0.2)
OSHA direct notification	1 (0.2)
Other	2 (0.4)
Missing	27 (5.7)
**Total**	**470 (100)**

**Abbreviations:** BSEE = Bureau of Safety and Environmental Enforcement; NIOSH = National Institute for Occupational Safety and Health; OSHA = Occupational Safety and Health Administration; USCG = U.S. Coast Guard.

The completeness of select variables was observed (Table 2). All incidents in the FOG database included information describing the number of OGE worker fatalities. Location (i.e., state or exact or estimated coordinates) also was identified for all worker fatalities. A majority of worker fatalities included basin information. Event type was identified in 94.3% of fatalities, and phase of operation and NAICS code information were identified for 89.8% of fatalities. Certain variables unique to OGE were identified in approximately 85% of worker fatalities (e.g., activity, working alone, and working unobserved). Demographic data were less common, with 79.6% of worker fatalities identifying sex, 75.7% identifying age, and 39.1% identifying race.

**TABLE 2:** Percentage of completeness of selected variables — Fatalities in Oil and Gas Extraction database, 2014–2019

Variable	Percentage complete
Injuries to multiple people	100
Number of OGE fatalities	100
Year	100
State	100
Latitude	100
Longitude	100
Basin	99.6
Event type	94.3
Phase of operation	89.8
NAICS code	89.8
Activity	85.3
Lone worker	85.3
Working unobserved	85.3
Sex	79.6
Age	75.7
Contractor size	74.0
Occupation	53.8
Race and ethnicity	39.1
Years in oil field	26.2

**Abbreviations:** NAICS = North American Industry Classification System; OGE = oil and gas extraction.

### Worker and Incident Characteristics

**FIGURE 2:**
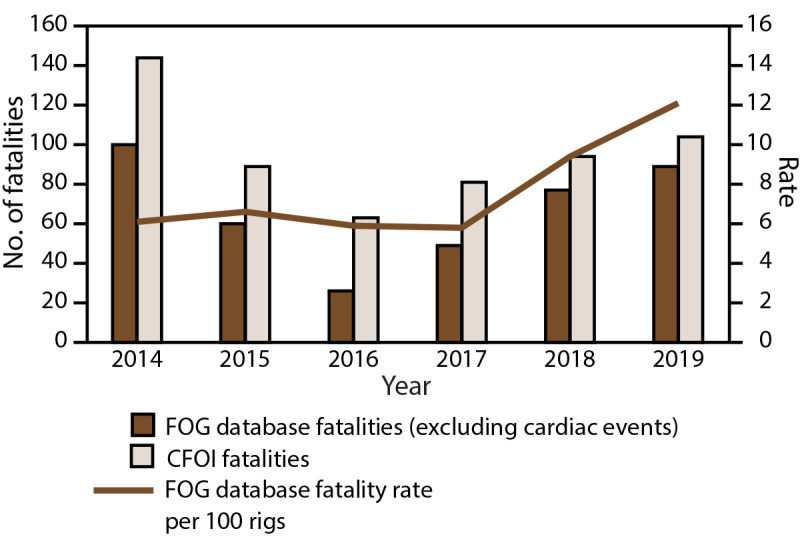
Number and rate* of fatalities among oil and gas extraction workers — Fatalities in Oil and Gas Extraction database and Census of Fatal Occupational Injuries, 2014–2019^†^ **Abbreviations:** CFOI = Census of Fatal Occupational Injuries; FOG = Fatalities in Oil and Gas Extraction. * No. of fatalities per 100 active rotary rigs. ^†^ N = 401 FOG fatalities; N = 575 CFOI fatalities.

The 470 worker fatalities identified in the FOG database during 2014–2019 included 401 occupational injury fatalities and 69 cardiac deaths (Table 3). The number of fatalities per year ranged from 30 in 2016 to 114 in 2019. The rate of FOG fatalities per 100 active rotary rigs ranged from 5.8 in 2017 to 12.1 in 2019. During 2014-2019, fewer OGE worker fatalities caused by occupational injury (excluding cardiac fatalities) were in the FOG database than in CFOI each year, ranging from a difference of 15.5% (2019) to 83.1% (2016). The percentage differences were the lowest during the final 2 years of data collection. The number of fatalities and fatality rate declined in 2016 and steadily increased in the years thereafter (Figure 2).

**TABLE 3:** Number, percentage, and rate* of oil and gas extraction worker fatalities — Fatalities in Oil and Gas Extraction database and Census of Fatal Occupational Injuries, 2014–2019

Year	FOG database fatalities No. (%)*	Rate of FOG database fatalities*	FOG database fatalities excluding cardiac events No. (%)^†^	CFOI fatalities No. (%)	Annual percentage difference of occupational fatalities, FOG database versus CFOI
2014	113 (24.0)	6.1	100 (24.9)	144 (24.0)	−36.1
2015	65 (13.8)	6.6	60 (15.0)	89 (15.5)	−38.9
2016	30 (6.4)	5.9	26 (6.5)	63 (11.0)	−83.1
2017	51 (10.9)	5.8	49 (12.2)	81 (14.1)	−49.2
2018	97 (20.6)	9.4	77 (19.2)	94 (16.3)	−19.9
2019	114 (24.3)	12.1	89 (22.2)	104 (18.1)	−15.5
**Total**	**470 (100)**	**N/A**	**401 (100)**	**575 (100)**	**−35.7**

**Abbreviations:** CFOI = Census of Fatal Occupational Injuries; FOG = Fatalities in Oil and Gas Extraction; N/A = not applicable.

* Per 100 active rotary oil rigs. Rates calculated using average annual rig counts from Baker Hughes (https://rigcount.bakerhughes.com/na-rig-count).

^†^ FOG database fatalities attributable to cardiac events were excluded for comparison purposes because fatalities resulting from cardiac events are not reported in CFOI.

**FIGURE 3:**
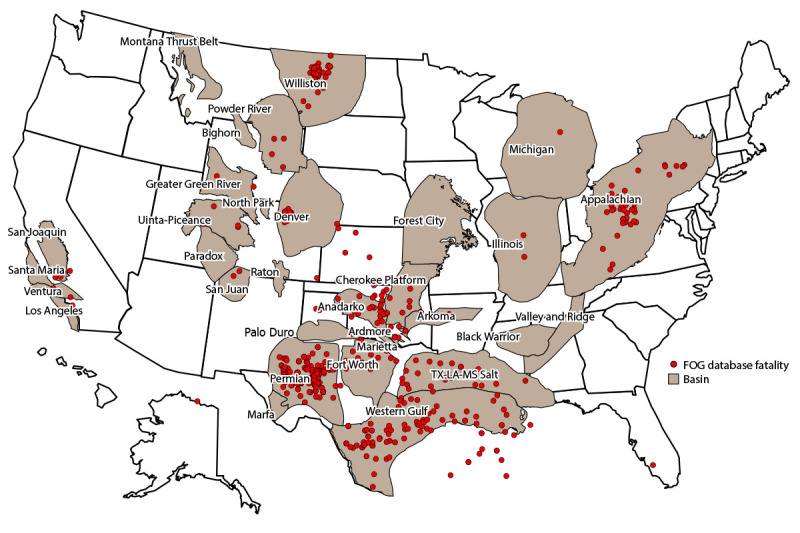
Oil and gas extraction worker fatalities* and U.S. basins — Fatalities in Oil and Gas Extraction database, 2014–2019 **Abbreviation:** FOG = Fatalities in Oil and Gas Extraction. N = 470.

Approximately one third of worker fatalities in the FOG database occurred in the Permian Basin (Table 4). A total of 15.7% of worker fatalities occurred in the Western Gulf Basin, followed by the Appalachian and Williston Basins with 9.1% and 8.5%, respectively (Figure 3).

**TABLE 4:** Number of worker fatalities, by basin — Fatalities in Oil and Gas Extraction database, 2014–2019

Basin	Fatalities No. (%)
Permian	148 (31.5)
Western Gulf	74 (15.7)
Appalachian	43 (9.1)
Williston	40 (8.5)
TX-LA-MS Salt	27 (5.7)
Anadarko	23 (4.9)
Denver	16 (3.4)
Fort Worth	13 (2.8)
Arkoma	10 (2.1)
Cherokee Platform	6 (1.3)
San Joaquin	6 (1.3)
Ardmore	5 (1.1)
Uinta-Piceance	5 (1.1)
Powder River	4 (0.9)
Greater Green River	2 (0.4)
Illinois	2 (0.4)
Palo Duro	2 (0.4)
San Juan	2 (0.4)
Los Angeles	1 (0.2)
Michigan	1 (0.2)
Ventura	1 (0.2)
N/A*	37 (7.9)
Unknown^†^	2 (0.4)
**Total**	**470 (100)**

**Abbreviation:** N/A = not available.

* Location offshore or not in any identified basin territory.

^†^ Cannot determine basin based on address details.

The mean age of worker fatalities in the FOG database was 41.2 years (Table 5). A total of 3.6% of worker fatalities were among those aged <21 years, and 8.1% were among those aged ≥61 years. One fourth of worker fatalities were missing age information. The majority of worker fatalities were males, representing 98.7% of worker fatalities with available sex information. Race and ethnicity information was less commonly available (60.9% unknown). A total of 23.2% of worker fatalities in the FOG database were among White persons and 13.6% were among Hispanic or Latino persons.

**TABLE 5:** Number of oil and gas extraction worker fatalities (N = 470), by selected characteristics — Fatalities in Oil and Gas Extraction database, 2014–2019

Characteristic	Fatalities No. (%)
**Age, yrs (mean = 41.2; SD = 14.1)**	
<21	17 (3.6)
21–30	86 (18.3)
31–40	74 (15.7)
41–50	74 (15.7)
51–60	67 (14.3)
≥61	38 (8.1)
Unknown	114 (24.3)
**Sex**	
Female	5 (1.1)
Male	369 (78.5)
Unknown	96 (20.4)
Race and ethnicity*	
American Indian or Alaska Native	1 (0.2)
Asian	1 (0.2)
Black or African American	7 (1.5)
White	109 (23.2)
Hispanic or Latino	64 (13.6)
Other	2 (0.4)
Unknown	286 (60.9)

* Persons of Hispanic or Latino (Hispanic) origin might be of any race but are categorized as Hispanic; all racial groups are non-Hispanic.

**FIGURE 4:**
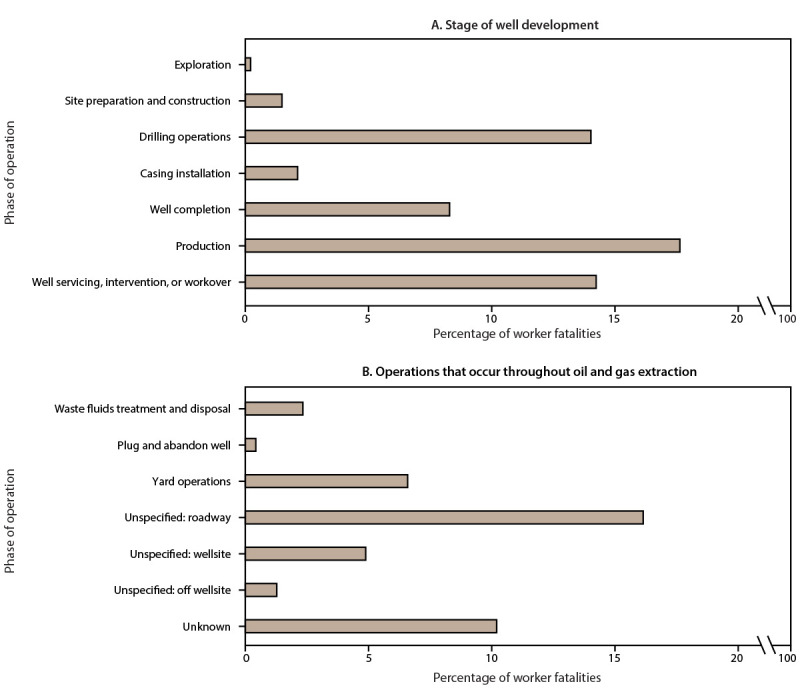
Percentage of oil and gas extraction worker fatalities,* by phase of operation — Fatalities in Oil and Gas Extraction database, 2014–2019 N = 470.

The most common phases of operation in worker fatalities were production (17.7%) and unspecified: roadway (16.2%), followed by well servicing, intervention, or workover (14.3%) and drilling operations (14.0%) (Figure 4) (Supplementary Table 3, https://stacks.cdc.gov/view/cdc/131261). The phase of operation could not be identified in 10.2% of worker fatalities.

Of all worker fatalities, 60.4% were among well servicing company workers, followed by drilling contractors (17.9%) and operators (5.1%) (Table 6). A total of 30 (6.4%) worker fatalities were among workers in industries other than industries with traditional OGE NAICS codes. Vehicle incidents were responsible for 26.8% of worker fatalities, a majority of which occurred on the roadway (77.0%). Contact injuries were the next most common event type (21.7%). Of 69 cardiac events (14.7%), a majority of these (81.2%) had no known work exposure. Thirteen cardiac events had a possible work exposure identified (2.8% of all worker fatalities). In total, 10.0% of worker fatalities involved exposures (i.e., harmful substances, work exposures possibly related to a cardiac event, alcohol or drug overdose, or environmental exposures). The most common activities involved in worker fatalities were repair or maintenance (13.4%), travel in a light duty vehicle (13.0%), and working with drilling fluids or tubulars (11.3%). Approximately one fifth of worker fatalities involved persons who were working alone at the time of the incident. Twenty worker fatalities in the FOG database occurred offshore (4.3%).

**TABLE 6:** Number of oil and gas extraction worker fatalities (N = 470), by selected industry characteristics — Fatalities in Oil and Gas Extraction database, 2014–2019

Characteristic	Fatalities No. (%)
**Industry group**	
Operators (NAICS 211)	24 (5.1)
Drilling contractors (NAICS 21311)	84 (17.9)
Well servicing companies (NAICS 213112)	284 (60.4)
Other	30 (6.4)
Unknown	48 (10.2)
**Event type**	
Vehicle incidents	126 (26.8)
Roadway	97 (20.6)
On-site or other location*	29 (6.2)
Contact injuries^†^	102 (21.7)
Explosions	68 (14.5)
Combustion or fire	60 (12.8)
Pressure	8 (1.7)
Cardiac event: no known work exposure	56 (11.9)
Exposures	47 (10.0)
Harmful substance	16 (3.4)
Cardiac event: possible work exposure	13 (2.8)
Other^§^	18 (3.8)
Falls^¶^	23 (4.9)
Electrocution	16 (3.4)
Intentional act	5 (1.1)
Unknown	27 (5.7)
Activity**	
Repair or maintenance^††^	63 (13.4)
Travel: light duty vehicle^§§^	61 (13.0)
Working with drilling fluids or tubulars^¶¶^	53 (11.3)
Transport (and transfer): fluids	42 (8.9)
Material handling***	39 (8.3)
Wellhead and well control equipment activities^†††^	32 (6.8)
Transport: equipment, proppant, supplies, or other types of cargo^§§§^	28 (6.0)
Downhole activities: casing, well stimulation, and control^¶¶¶^	24 (5.1)
Housekeeping and cleaning****	23 (4.9)
Rigging up or down	22 (4.7)
Break or rest	18 (3.8)
Equipment assembly or dismantle	18 (3.8)
Tank opening, gauging, or sampling	14 (3.0)
Hot work and welding	13 (2.8)
Transport: unspecified cargo	11 (2.3)
Travel: aircraft, water vehicle, or other^††††^	10 (2.1)
Pressure pumping	9 (1.9)
Wellsite construction or upkeep	8 (1.7)
Chemical handling	7 (1.5)
Lease operation	7 (1.5)
Spotting	6 (1.3)
Office and administrative activities	5 (1.1)
Training	5 (1.1)
Well cleanout	5 (1.1)
Wireline and slickline activities	4 (0.9)
**Working alone**	
Yes	101 (21.5)
No	300 (63.8)
Unknown	69 (14.7)
**Offshore**	
Yes	20 (4.3)
No	450 (95.7)

**Abbreviation:** NAICS = North American Industry Classification System.

* Includes vehicle incident: on-site and vehicle incident: other location.

^†^ Includes the three types of contact injuries (i.e., caught between or crushed, struck by, and struck by falling object).

^§^ Includes exposure: alcohol or drug overdose, exposure: environmental, and exposure: undetermined (possible work exposure).

^¶^ Includes fall: from height and fall: same level (i.e., slip or trip).

** Activities sum to >470 because each incident is assigned as many activities as needed to adequately characterize the event.

^††^ Includes the seven types of repair or maintenance (i.e., equipment, flowlines, rig, separation equipment, tanks, tubulars, and vehicle) as well as tank refurbishment and custom fabrication.

^§§^ Includes the three subcategories of light duty vehicle travel (i.e., nontraditional, on-duty, and unknown).

^¶¶^ Includes the four types of drilling, pulling, or running tubulars (i.e., lay down or pick-up, make up or break-out, other, and racking back) as well as drilling fluid mixing and pumping.

*** Includes material handling: manual and material handling: powered equipment.

^†††^ Includes well control equipment activities and wellhead or pumping unit activities.

^§§§^ Includes transport: equipment, proppant, or supplies hauling and transport: other types of cargo.

^¶¶¶^ Includes cementing, coiled tubing activities, fishing, hot oiling, perforating, plug drill out, pressure testing, snubbing, swabbing, and well testing or logging.

**** Includes the five types of housekeeping and cleaning (i.e., equipment, other, rig, tanks, and unspecified).

^††††^ Includes travel: aircraft, travel: other, and travel: water vehicle.

**FIGURE 5:**
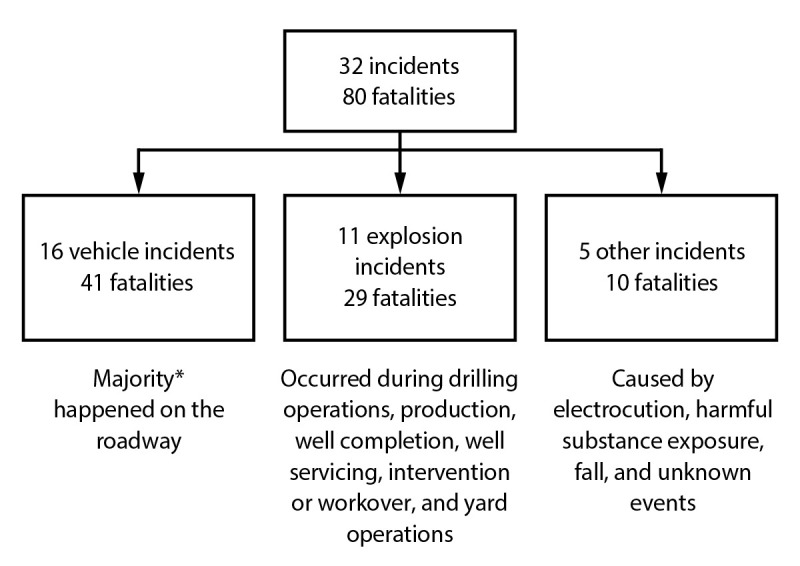
Multifatality incidents and fatalities — Fatalities in Oil and Gas Extraction database, 2014–2019* * Vehicle incidents: 81.3%; vehicle fatalities: 85.4%.

Of the 470 worker fatalities, 80 (17.0%) resulted from multifatality incidents (Figure 5). These fatalities resulted from 32 total incidents. Vehicle incidents were responsible for one half of the multifatality incidents, resulting in 41 worker fatalities. Explosions caused 11 incidents, resulting in 29 worker fatalities. The remaining five incidents (i.e., electrocution, exposure to harmful substance, fall, or unknown event) resulted in two fatalities each.

### Texas Department of Transportation Crash Records Information System

The pilot program using Texas Department of Transportation CRIS motor vehicle crash records identified 56 worker fatalities resulting from crashes during 2017–2019 not included in the 470 worker fatalities (Table 7). Approximately 60% of crash fatalities were workers employed by well servicing companies. Approximately three fourths of CRIS fatalities were collisions with other vehicles, followed by 19.6% noncollision incidents and 8.9% collisions with objects other than a vehicle. Four crashes were multifatality incidents, resulting in two fatalities each. One half of CRIS fatalities involved a pickup truck and 41.1% involved a semitrailer, tractor trailer, or trailer truck. In 51.8% of fatalities identified through CRIS, a seat belt was not worn. A total of 21.4% of all CRIS fatalities involved the person being ejected from the vehicle. One half of the CRIS fatalities occurred between the hours of midnight to 9:00 a.m., followed by approximately one fourth occurring between 9 a.m. and 5 p.m. and the remaining 20% occurring between 5 p.m. and midnight.

**TABLE 7:** Number of oil and gas extraction worker fatalities (N = 56) identified through Texas Department of Transportation Crash Records Information System, by selected characteristics — Fatalities in Oil and Gas Extraction database, 2017–2019

Characteristic	Fatalities No. (%)
**Industry group**	
Operators (NAICS 211)	3 (5.4)
Drilling contractors (NAICS 21311)	2 (3.6)
Well servicing companies (NAICS 213112)	36 (64.3)
Other	9 (16.1)
Unknown	6 (10.7)
**Age group, yrs**	
21–30	16 (28.6)
31–40	11 (19.6)
41–50	14 (25.0)
51–60	12 (21.4)
≥61	3 (5.4)
**Crash type**	
Collision with other vehicle	40 (71.4)
Noncollision incident	11 (19.6)
Collision with object other than vehicle	5 (8.9)
**Vehicle type**	
Pickup truck	28 (50.0)
Other semitrailer, tractor trailer, or trailer truck	23 (41.1)
Water hauler	3 (5.4)
Automobile	1 (1.8)
Van	1 (1.8)
**Seat belt used**	
No	29 (51.8)
Yes	21 (37.5)
Unknown	6 (10.7)
**Ejected from vehicle**	
No	43 (76.8)
Yes	12 (21.4)
Unknown	1 (1.8)
**Time of day**	
Midnight–9:00 a.m.	30 (53.6)
9:00 a.m.–5:00 p.m.	15 (26.8)
5:00 p.m.–midnight	11 (19.6)

**Abbreviation:** NAICS = North American Industry Classification System.

## Discussion

This report is the first to describe the FOG database creation and methods with an overview of worker fatalities during 2014–2019. The number of worker fatalities in the FOG database was less than the number of OGE fatalities in CFOI, which might be attributed to CFOI having access to more data sources for case identification compared with the FOG database. The inclusion criteria between databases also differs. The FOG database included 30 worker fatalities that were OGE related but assigned a NAICS code other than what CFOI identifies as OGE related. Although the purpose of CFOI is to collect a census of fatalities for all industries, the FOG database collects detailed OGE industry-specific information on fatally injured workers who might not typically be classified as working in OGE.

The FOG database also includes worker fatalities attributable to cardiac events and crashes during commutes, both of which are excluded from CFOI. Long commutes are common among OGE workers (*5*) and capturing fatalities occurring during commutes is vital to fully understand OGE worker fatalities. Cardiac events beginning at work are also important to investigate among OGE workers because exposure to certain chemicals or toxic substances used during OGE activities can mimic or induce cardiac events (*30*) (Supplementary Appendix 2, https://stacks.cdc.gov/view/cdc/131261). Among the 69 cardiac events, 13 workers had a possible work exposure. Cardiac events also can follow extreme physical exertion, which is common for certain OGE occupations. Working alone is an additional characteristic of OGE work arrangements that is important to consider relative to cardiac events and all fatalities, because 21.5% of worker fatalities in the FOG database were OGE workers working alone. The majority of worker fatalities in FOG also occurred in rural counties (FOG database, unpublished data, 2023), further complicating and potentially delaying emergency response for OGE workers involved in a cardiac or injury incident. Lone worker programs, medical screening, and enhanced exposure controls might be beneficial for the OGE industry (*31*).

The rate of FOG fatalities per 100 active rotary rigs was calculated to evaluate the association between the number of fatalities each year and the level of industry activity, which has shown contrasting relations in previous years (*4*,*32*). Although equivalent numbers of worker fatalities were identified in the FOG database for 2014 and 2019, the corresponding fatality rate for 2019 was approximately twice that of 2014 because fewer rotary rigs were active in 2019. Although the increased fatality rates in 2018 and 2019 compared with earlier years might suggest less safe working conditions resulting from high demand in the industry, the increased rates also might result from increases in drilling efficiency, which leads to lower numbers of active rotary rigs. Both the number of wells drilled and the number of active rotary rigs have decreased in recent years, whereas increases in production have reached new records (*33*).

Accounting for approximately 40% of U.S. oil production (*34*), the Permian Basin had the largest proportion of worker fatalities in the FOG database (31.5%). Worker fatalities in the FOG database are largely concentrated in Texas, which is the top oil- and gas-producing state with the majority of active rotary rigs and OGE workers (*9*,*28*,*35*).

Worker demographics and industry characteristics were representative of what is known about the U.S. OGE worker population. Among OGE workers included in the FOG database, the mean age was 41 years, and 14% of workers had Hispanic or Latino ethnicity, which is similar to national estimates of OGE workers identifying a median age of 42.9 years, and 14.6% with Hispanic or Latino ethnicity in 2019 (*36*); however, a large proportion of data related to demographics in the FOG database was missing. The higher proportion of fatally injured well servicing company workers compared with drilling contractors and operators was similar to the 57.1% of well servicing company workers in the OGE industry in 2019 and consistent with an earlier analysis of CFOI data that OGE well servicing contractors had the highest number of worker fatalities (*4*,*9*).

Consistent with previous research of OGE fatalities (*4*), vehicle incidents were the most common event type among worker fatalities in the FOG database. Analysis of the additional fatalities identified through CRIS motor vehicle crash records during 2017–2019 indicated that approximately one half of the CRIS fatalities occurred in pickup trucks and approximately one half of the fatally injured workers were not wearing seat belts. Previous research of OGE fatalities has also identified low levels of seat belt use (*13*,*37*), indicating that employer interventions to increase seat belt use are needed in the industry. Although the CRIS pilot study successfully identified 56 additional fatalities that were not identified in the FOG database, future research to analyze motor vehicle crashes in the OGE industry might benefit from exploring other data sources that do not require the time and resources needed to review the CRIS database.

Vehicle incidents also were responsible for one half of the multifatality incidents in the FOG database, the majority of which occurred on a roadway. Researchers were not able to deduce why multifatality vehicle incidents might be occurring because information on contributing factors (e.g., distraction and fatigue) is limited. However, this observation serves as a reminder that research investigating OGE worker fatalities needs to consider the risks for substantial driving exposure and long commutes, which are common among OGE workers (*5*). Other multifatality incidents in the FOG database were caused by explosions, electrocutions, exposure to harmful substances, and falls. One study analyzing multifatality work-related incidents in the United States during 1995–2010 similarly found that approximately one half of fatalities were caused by transportation accidents, followed by fires and explosions and assaults and violent acts (*38*). Certain multifatality incidents caused by explosions might receive more media coverage and response from the industry (e.g., the Deepwater Horizon rig explosion in 2010) and might lead to additional industry regulations and enforcement of these regulations (*39*). The largest proportion of multifatality incidents result from vehicle incidents and often go unnoticed.

Only 4.3% of worker fatalities in the FOG database occurred during offshore operations. A previous analysis of CFOI data during 2003–2010 identified an average of 16 offshore OGE fatalities per year (*14*). In contrast, 20 offshore fatalities were identified in the FOG database during 2014–2019. This apparent decline in offshore fatalities might have resulted from the reform in offshore regulations in response to the Deepwater Horizon rig explosion or a decline in helicopter incidents resulting from new technology and safer practices (*40*). In addition, BSEE reported only 12 fatalities from fiscal year 2014 through calendar year 2019 (*41*). This variation between the total offshore fatalities in the FOG database and those reported by BSEE is likely because of BSEE reporting requirements, which do not include fatalities occurring while workers are in transport to offshore facilities (*42*). NIOSH researchers consider traveling to offshore facilities a work-related activity because of the remoteness of offshore OGE work and include these fatalities in the FOG database.

### Contributions to Industry

The initial findings from the FOG database highlighted acute exposures to hydrocarbon gases and vapors among OGE workers that were previously unreported (Supplementary Appendix 2, https://stacks.cdc.gov/view/cdc/131261). These range from special topic reports and scientific articles to hazard alerts and videos. Identification of unreported acute exposures to hydrocarbon gases and vapors among workers who open tank hatches led the American Petroleum Institute (API) to develop a new safety standard in 2016. This standard offers safer methods for measuring crude oil without opening the tank hatch to protect workers from exposure to hydrocarbon gases and vapors (*43*). The standard was also adopted by the U.S. Department of the Interior’s Bureau of Land Management through an update of rules for oil measurement on federal and Indian gas leases. The update no longer requires companies to obtain variances (i.e., exceptions) to use alternative measurement methods, making it easier to implement safer practices.

Additional NIOSH publications highlighted selected periods of FOG data as well as hazard alerts and fact sheets on various topics. Hazard alerts and fact sheets were published in collaboration with OSHA, NIOSH, and the National Service, Transmission, Exploration and Production Safety (STEPS) Network Alliance and describe known hazards along with recommendations for employers and workers on how to improve safety for workers (*44*). Hazard alerts have been distributed through the National STEPS Network and through contractor management systems (e.g., Veriforce [formerly PEC Safety]; https://veriforce.com) to thousands of workers. Additional recommendations for medical professionals and medical examiners and coroners were provided by occupational medicine physicians and researchers to help identify signs and symptoms of exposure to hydrocarbon gases and vapors and oxygen-deficient atmospheres in OGE workers (*30*). Three scientific articles highlight three trends in OGE fatalities identified through the FOG database (i.e., cardiac events, heat-related illness, and substance use) and provide employer considerations to mitigate risks for fatality. Findings based on FOG data have been presented to multiple researchers, industry safety and health groups, and other industry leaders. Groups who have received data from the FOG database include API, IADC, the Association of Energy Service Companies (now Energy Workforce & Technology Council), the American Society of Safety Professionals, and the Permian Road Safety Coalition.

### Surveillance System Strengths and Challenges

The creation of the FOG database and the first 6 years of data illustrate that the database is a valuable resource for identifying safety and health trends and emerging issues among OGE workers. Benefits of an industry-specific surveillance system such as the FOG database include the ability to identify emerging hazards, temporal trends, and risk factors that might be unique to the industry and to build relationships with industry partners with the mutual goal of promoting worker safety and health. The FOG database also helps researchers to better identify groups of workers who are at increased risk for injury. Emerging safety and health issues among OGE workers were identified through analysis of the FOG database (e.g., exposure to hydrocarbon gases and vapors and fatalities caused by heat-related illness, substance use, and cardiac events) (Supplementary Appendix 2, https://stacks.cdc.gov/view/cdc/131261). The FOG database includes detailed information describing the circumstances of each incident and the fatally injured worker while also capturing key variables that can be collapsed to allow for broader epidemiological analyses. The narratives describing each fatality also provide data that have been valuable to industry partners.

Certain challenges exist when maintaining an industry-specific surveillance system. The FOG database case identification and data collection process is labor intensive and requires multiple researchers with substantial knowledge of the OGE industry and sustained partnerships with organizations from which case information can be requested. Approximately one half of worker fatalities in the FOG database originally were identified through reports sent from OSHA to NIOSH, which might have occurred because OSHA does not report on fatalities involving self-employed workers or on a majority of transportation incidents. Identifying a large portion of worker fatalities required researchers to search other databases, examine newsletters and other alerts, and rely on contacts in the industry.

The source document collection and case review processes are time consuming and often result in documents lacking detail or received long after the incident has occurred. Periodic follow-up with OSHA area offices often is necessary until investigations are finished and all complete case files are obtained. Once received, researchers must review hard-copy OSHA case files that often exceed 100 pages to extract information relevant for inclusion in the FOG database. OSHA case files often do not contain the level of detail to identify all factors contributing to the fatality. Relevant information might be missing because OSHA investigations are conducted by compliance safety and health officers whose purpose is to determine whether OSHA safety and health standards were violated. If so, the officer must justify which standard is violated, to what degree, and issue a citation and recommend a fine. OSHA does not maintain a standard for OGE work; thus, citations issued by officers often are based on OSHA’s General Duty Clause, which lacks considerable OGE context. The purpose of the FOG database is to serve as a public health prevention tool by monitoring trends in the industry and identifying emerging issues. As a result, this lack of context in OSHA’s General Duty Clause often omits details important for prevention. For example, long work hours and fatigue are issues common among OGE workers; however, OSHA case files often do not report how long the fatally injured person was working before the incident or any details regarding work schedules.

When the worker fatality is not investigated by OSHA, researchers spend substantial time locating crash reports, autopsies, and other source documents from local and state governments. This process leads to delays in access to timely data and additional time spent extracting information from data sources that are not focused on public health surveillance. FOG data are not being collected beyond 2019 because of these challenges and the end of grant funding. Additional funding and access to OSHA data systems could allow for data collection in the future. Missing data also are common in source files. In addition, autopsies and toxicology reports are not always conducted or made available to researchers. When reports are available, certain toxicology tests that are relevant to exposures in the OGE industry are not always performed on workers, making it difficult to estimate the true prevalence of exposure to hazardous substances among OGE workers (*31*).

Demographic data in the FOG database were less complete than for a majority of OGE industry-specific variables, particularly for fatalities involving cardiac events. This observation might be because of the inclusion of fatalities in reports provided by OSHA beginning in 2018 that were deemed not work related and therefore lacked details other than a brief summary of the incident. More complete information useful for prevention could be obtained from OSHA case files through increased collaboration with OSHA investigators, medical examiners, and other persons performing death investigations (e.g., systematic collection of demographic information about the worker death could be collected at the time of employer reporting). In addition, when an OSHA compliance officer investigates a fatally injured OGE worker, further information related to the working conditions (e.g., work hours, schedule, and activities performed) would be beneficial.

## Limitations

The findings in this report are subject to at least three limitations. First, data for self-employed workers are limited. Because OSHA does not investigate these worker fatalities, they do not appear in any of the OSHA case identification sources; therefore, findings from the FOG database might underestimate the number of fatalities. Second, fatalities resulting from work-related chronic illness cannot be identified in the FOG database because the latency between exposure and diagnosis makes it difficult to determine whether an illness was work related. In addition, there is a lack of data sources that document these types of illnesses. Finally, motor vehicle crashes that occur on public roads and highways are underreported in the FOG database. Data sources identifying this type of incident are limited because a majority of transportation incidents are outside of OSHA’s jurisdiction. The pilot program review crash records from CRIS was an attempt to improve information collection about roadway motor vehicle crashes from a state agency, but it is too labor intensive for continued inclusion in the FOG database. In addition, only crashes involving workers in company vehicles were included, so it is possible that crashes involving workers commuting to the well site or during nonstandard commutes in their personal vehicles were not identified.

## Conclusion

Maintaining an industry-specific worker fatality surveillance system is labor intensive yet yields numerous benefits for industry, occupational safety and health practitioners, and government researchers and is useful to guide interventions to prevent worker injuries and fatalities. The FOG database contained 470 OGE worker fatalities during 2014–2019. Researchers used the FOG database to identify emerging hazards and trends in the OGE industry, such as exposure to hydrocarbon vapors and gases and fatalities resulting from heat, cardiac events, and substance use. Limitations of the database include missing data, delays in access to timely data, and the need for researchers with substantial knowledge of the OGE industry. Additional analysis of worker fatalities during 2014–2019 in the FOG database might be conducted in response to industry needs and researcher priorities. Continued surveillance of worker fatalities in the OGE industry can help identify new safety and health hazards, especially as demand for oil and gas continues and characteristics of the OGE workforce and technology constantly change. Robust safety and health management systems, a positive workplace safety culture, and collaboration among government, academic institutions, and industry partners are essential to improving worker safety.

## Footnotes

* 45 C.F.R. part 46, 21 C.F.R. part 56; 42 U.S.C. §241(d); 5 U.S.C. §552a; 44 U.S.C. §3501 et seq.

† Google Alert search criteria: [(“drilling site”|”drilling rig”|”oil and gas”|”oil rig”|”oilfield”|”production rig”|”production site”|”pump site”|”well site”) (fatality|~death|deceased|accident|injury|injured|”killed”|”kills”)].
